# A pilot randomised controlled trial evaluating mini and conventional implant retained dentures on the function and quality of life of patients with an edentulous mandible

**DOI:** 10.1186/s12903-017-0333-1

**Published:** 2017-02-15

**Authors:** Sarra Jawad, Craig Barclay, William Whittaker, Martin Tickle, Tanya Walsh

**Affiliations:** 1 Department of Restorative Dentistry, University Dental Hospital of Manchester, Higher Cambridge Street, Manchester, M15 6FH UK; 2 Division of Population Health, Health Services Research and Primary Care, School of Health Sciences, University of Manchester, Manchester, UK; 3 Division of Dentistry, Faculty of Biology, Medicine and Health, University of Manchester, Manchester, UK

**Keywords:** Dental implant, Edentulous, Complete overdenture, Randomised controlled trial, Mini implant, Quality of life, Masticatory efficiency

## Abstract

**Background:**

Total tooth loss (edentulism) can be a debilitating condition, impacting on ability to chew, speak and interact with others. The most common treatment is with complete removable dentures, which may be successful, but in the lower jaw, bone resorption that worsens over time makes denture-wearing difficult. Two dental implants in the mandible to retain the lower denture has been advocated as the gold standard of treatment, but has not been universally provided due largely to financial constraints and also patient fear. Mini implants (MI) are cheaper and less invasive than conventional implants (CI), but may not have equivalent longevity. Therefore, it is unknown whether they represent a cost-effective treatment modality over time. The aim of this pilot randomised controlled trial was to assess the feasibility of carrying out a trial on this cohort of patients, and to inform the study design of a large multicentre trial.

**Methods:**

Forty-six patients were randomly allocated to receive either two mini implants or two conventional implants in the mandible to retain their lower dentures. Quality of life (QoL) questionnaires, pain and anxiety scores, and an objective “gummy jelly” chewing test were carried out at multiple timepoints, along with detailed health economics information. Implants were placed one-stage, and an early loading protocol was utilised. Patients were reviewed 8 weeks post-placement, and finally at 6 months. Implant failure, recruitment and retention rates were recorded and analysed.

**Results:**

The pilot study demonstrated that it is possible to recruit, randomise and retain edentulous (mainly elderly) patients for an implant trial. We recruited to target and retention rates were acceptable. The large number of questionnaires was onerous for participants to complete, but the distribution of scores and feedback from participants helped inform the choice of primary and secondary outcomes in a full trial. The chewing test was time-consuming and inconsistent. Implant failure rate was low (1/46). The data on indirect costs gathered at every visit was viewed as repetitive and unnecessary, as there was little or no change between visits.

**Conclusions:**

The pilot study has shown that acceptable recruitment and retention rates are achievable in this population of patients for this intervention. The results provide valuable information for selection of outcome variables and sample size calculations for future trials.

**Trial registration:**

(ISRCTN): 87342238 Trial registration date: 05/07/2013.

**Electronic supplementary material:**

The online version of this article (doi:10.1186/s12903-017-0333-1) contains supplementary material, which is available to authorized users.

## Background

The World Health Organisation (WHO) considers edentulism (total tooth loss) a physical disability [1, 2]. The majority of edentulous patients are able to chew their food with complete dentures but over time, the lower jaw becomes resorbed and there is less bone to retain the lower denture. This makes it more difficult to retain the denture which causes problems for the denture-wearer, such as difficulties in eating and speaking, which may lead to a change in lifestyle, as those affected become embarrassed to socialise and eat with friends [3]. Furthermore, their inability to chew results in poorer food choices [4, 5] with many opting for highly calorific softer foods that are easier to eat. This undoubtedly affects their nutrition and thus general health. All of these factors have been shown to impact greatly on a patient’s quality of life [6].

The dental literature is awash with papers showing significant improvements in quality of life in edentulous patients who have two dental implants placed in their lower jaw to secure their lower dentures [6–15]. Indeed there are national and international consensus statements that this treatment modality should be the first line of treatment for patients with an edentulous mandible [16, 17]. However, this treatment is costly for both individual patients and health services around the world to provide. Furthermore, surgery to place implants is invasive [18], and this can pose a barrier to treatment even when provided free of charge [19, 20]. Edentulous patients are often older people (over 65 years) and may have significant bone resorption in their lower jaw and complex medical histories, which may affect their suitability for implant treatment.

Mini dental implants have been in use for the last 12 years [21–36] and offer a number of advantages over conventional implants: they have a smaller diameter (<2.4 mm) and are often made of a titanium alloy (Ti 6Al-4 V ELI) as opposed to commercially pure titanium (type 4) used in conventional dental implants. They are often placed using a minimally invasive technique, resulting in less post-operative pain [37]; and are therefore approximately a quarter of the cost of current conventional alternatives [21, 22]. These advantages need to be considered against a potentially higher failure rate [38] when compared to conventional implants, which have an impressively high survival rate over 10 years [15], and possibly a need for more intensive on-going maintenance [39].

There is a lack of high quality evidence of the effects of mini implants compared to conventional implants in retaining mandibular complete dentures [39, 40]. The primary aim of this study was to assess the feasibility of conducting a large surgical randomized controlled trial of mini versus conventional implants in a population of patients who can benefit from an implant- retained lower denture. The identified key areas of uncertainty to be explored in this trial focused upon the processes of recruitment and retention of participants, choice of clinical, quality of life and cost outcome measures and their method of capture. The objectives were specifically to determine:

- The proportion of referred patients that were eligible to participate, consented to the trial and provided data at all time-points;
- Whether masticatory functional ability could be measured using a validated ‘gummy jelly’ chewing test;
- The completeness and variability of using different instruments to collect data on pain, QoL, implant failure; and
- How to collect and measure costs

## Methods

### Design and eligibility criteria

The pilot trial was a two-arm parallel group randomised controlled pilot trial. Full ethical approval was obtained from the National Health Service Health Research Authority, National Research Ethics Service, Research Ethics Committee (ref: REC 13/NW/0384), and the study clinicians collected informed consent. All edentulous patients experiencing difficulties with their lower dentures, referred to the University Dental Hospital of Manchester or from internal referrals from hospital consultants, were initially screened for eligibility from the referral letters. Potentially eligible participants were booked onto consultation clinics for full medical history and clinical examination. Patients with an edentulous mandible, with residual mandibular ridge Cawood and Howell Class V or VI [41] with continued difficulties eating, even after the provision of new dentures, were eligible to participate. Patients with a history of bisphosphonate therapy or implant treatment, requiring sedation or general anaesthetic for implant placement, smokers, or patients unable to maintain adequate levels of oral hygiene were excluded.

### Randomisation

The Clinical Trials Unit centrally randomised patients to one of two treatment groups with a 1:1 allocation ratio using random permuted blocks. A study clinician enrolled participants onto the trial and assigned participants to interventions according to the randomisation. The clinician performing the implant placement and health economist could not be blinded to treatment allocation; the research nurse (who collected masticatory efficiency and patient rated outcome data) and the trial statistician were blinded.

### Treatment procedures

All patients were given an oral loading dose of 2 mg Amoxicillin (or 600 mg Clindamycin if allergic to penicillin) as per best current evidence [42]. All patients were asked to rinse for 60 s with Chlorhexidine (0.2%) mouthwash (with both dentures removed) and given local anaesthetic using 2% lignocaine + 1:80,000 adrenaline infiltrated into the anterior mandible.

#### Mini-implant (MI)

Two mini implants (3 M® 2.1 mm diameter, 10 mm length one-piece implant with a square collar and ball abutment) were placed transmucosally (flapless) in the interforamina region of the edentulous mandible.

#### Conventional implant (CI)

Two conventional (ASTRA TECH® Osseospeed 3 mm diameter, 11 mm length) implants were placed in the interforamina region of the edentulous mandible. These were placed after raising soft tissue flaps and drilling directly into bone. Ball abutments were placed on the conventional implants in a one-stage surgery approach to mimic the mini-implant attachment system.

Following surgery, all patients were given oral and written post-operative instructions and advised not to wear their lower dentures for 1 week. All patients were given 24 × 500 mg Paracetamol tablets to take as required. At 1 week postoperatively participants’ lower dentures were hollowed out chair-side so they could fit over the exposed ball abutments. Therefore, lower dentures could be worn but the implants were not directly loaded. At the 2-month review appointment an impression was taken of the implant ball abutments using a polyether impression material (Impregum™ Penta™ 3 M ESPE) in the patient’s lower complete denture. The denture was retrofitted with stud attachments on the same day by dental laboratory technicians using heat-cured acrylic.

### Outcomes

Multiple outcome measures were assessed in accordance with the objectives of the study and as per the schedule below (Table 1). The primary outcomes of this study were feasibility outcomes. The feasibility of a main trial using the same study design and protocol was assessed according to the objectives of the pilot trial: recruitment and retention of trial participants; use of outcome measures and collection of cost data.

**Table 1:** Outcome measures and timing of measurement

	Baseline	At operation	24 h	1 week	2 months	6 months
Anxiety: Modified Dental Anxiety Scale [64]	X					
Quality of Life: Oral Health Impact- Profile-EDENT [53, 65] Euroqol-5D(EQ-5D) [44, 66] SF-12 [67]	X				X	X
Satisfaction with dentures: The University of Newcastle ‘Assessment of Prosthesis’ questionnaire	X				X	X
Masticatory function: ‘gummy jelly’ chewing test	X				X	X
Pain: Visual Analogue Scale (VAS) or Verbal Rating Scale (VRS) (0–10)		X	X	X		
Use of analgesics (professionally and self prescribed) and antibiotics	X		X	X	X	X
Direct costs-staffing^a^		X				
Direct costs-equipment^a^		X				
Direct costs-consumables^a^		X				
Indirect costs^a^		X		X		
Healthcare utilisation^a^		X	X	X	X	X
Assessment of adverse events^a^		X	X	X	X	X
Assessment for possible implant failure				X	X	X

^a^data also to be collected at unscheduled visits

- The feasibility of recruitment was measured in terms of eligibility (the proportion of eligible patients from the number of screened patients), recruitment (the proportion of recruited patients from the number of eligible patients), and whether the required sample size could be met within the designated 12-month recruitment period. The success of patient retention was measured in terms of compliance with randomisation (the proportion of participants receiving their allocated treatment) completeness of follow-up (the proportion of patients providing 6 month follow up data). The denominator was the number of patients randomised.
- The feasibility of using masticatory efficiency as an effectiveness outcome was assessed as the number of ‘gummy jelly’ samples that were provided, and were able to be analysed.
- The appropriateness of the different outcome measures was assessed quantitatively in terms of proportion of completed questionnaires, and qualitatively in variation, absence of floor or ceiling effects and variability of responses over time.
- The feasibility and appropriateness of collecting cost information was assessed based on the proportions of completed patient questionnaires (patient costs) and completed health care costs, and the recording of unanticipated resource use.

Formal hypothesis testing for effectiveness was not be undertaken as the aim of a pilot trial is not to assess effectiveness and as such the study is underpowered for this purpose.

### Sample size

As this was a pilot trial a formal sample size calculation was not appropriate. We estimated that of those patients screened and meeting the inclusion criteria, 50% would agree to participate. A total of 44 patients (22 in each arm) would have a 95% confidence interval ranging from 35% to 65% of patients who will agree to participate. A recruitment target was set at 44 patients or 1 year of recruitment, whichever endpoint was reached soonest.

### Statistical analysis

It was not our intention to compare treatment groups and we did not test hypotheses. Cumulative and monthly (to assess season variation) recruitment figures were calculated, as were loss to follow up at 2 and 6 months post-surgery. Descriptive analyses were undertaken for a 12 month period (covering the period 6 months prior to the study start and the 6 months follow up) to identify the total number of referrals, the number and proportion of participants who attended for assessment, and the number and proportion of patients who required implants after fitting a new set of conventional complete dentures. Simple descriptive statistics involving calculation of ranges, frequency distributions and measures of central tendency and dispersion were used to assess the completeness and variability of clinical, quality of life and cost outcome measures collected at each time point.

The health economics analysis presents the resources recorded and their respective unit costs. Costs were calculated from the National Health Service (NHS) and social care provider perspective, secondary analysis includes costs from the patient perspective, and this approach approximates a societal perspective. The time trade-off (TTO) measurement for weighting the EQ-5D was used, as this is the recommended utility weight advocated by the National Institute for Health and Care Excellence NICE [43]. The TTO weights are obtained from Dolan (1997) [44]. The SF-12 was mapped onto the SF-6D (since the SF-6D has Health-Related QoL weights). SF-6D weights were obtained from Brazier and Roberts (2004) [45].

## Results

**Fig. 1:**
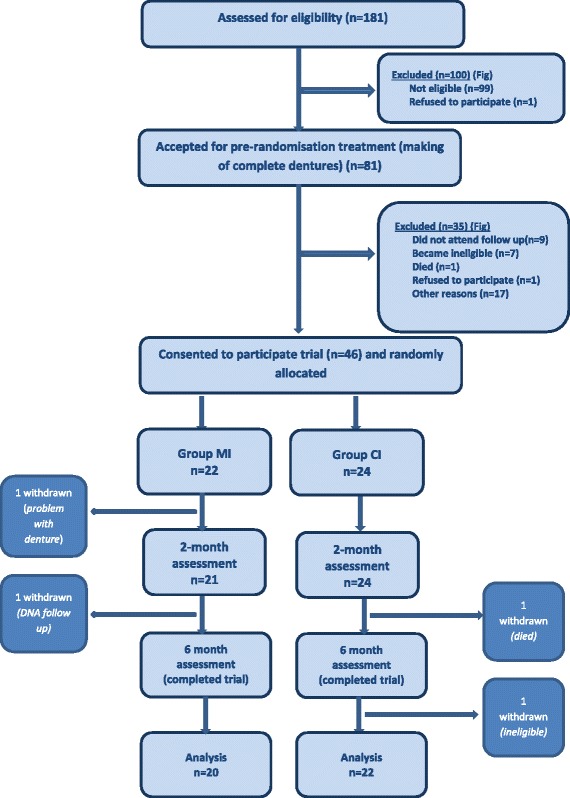
Flow diagram showing recruitment and retention

The recruitment and retention of participants from screening through to 6-month follow-up is shown in Fig. 1. Of note is that only 81/181 of those referred were eligible for the study, and of the 73 patients for whom new complete dentures were made, 12 were relatively satisfied with their new lower denture and declined further treatment. Furthermore, only four patients who were dissatisfied with their new denture did not want to be randomised, having a preference for mini-implants (three patients) or conventional implants (one patient). Of the 46 patients randomized 43 (93.5%) completed the trial.

**Fig. 2:**
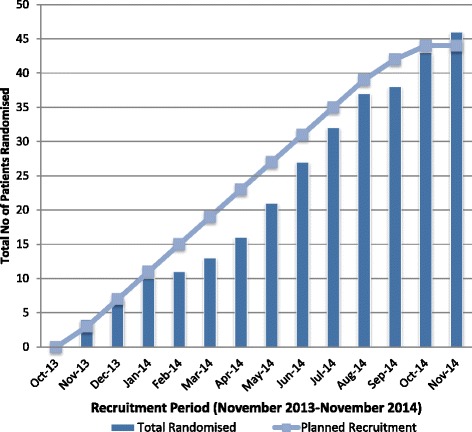
Bar chart showing actual recruitment versus projected recruitment (line graph)

Although the initial plan was to recruit a maximum of 44 participants or as many participants as possible in the planned 1-year recruitment period, in total 46 patients were recruited and randomised (further ethical approval was provided to recruit the additional participants). The median number of patients recruited per month was three and the monthly and cumulative recruitment figures are summarised in Fig. 2. The maximum number of patients recruited in a single month was six patients (June 2014); the minimum number of patients recruited was one (February 2014 and September 2014).

During the trial there was one protocol violation. During analysis it transpired that one patient was taking bisphosphonates a concomitant medication that that was not fully disclosed at recruitment. This patient was not included in the analyses. Table 2 shows the baseline characteristics of the trial participants.

**Table 2:** Summary statistics at baseline for patients analysed^a^

	Mini implants (*n* = 22)	Conventional implants (*n* = 23)	Total (*n* = 45)
Age	68.5 (9.5) 68.5, 63 to 74 52 to 88	68.1 (8.5) 71, 61 to 74 48 to 79	68.3 (8.9) 70, 62 to 74 48 to 88
Gender	10 male (45.5%)	9 male (39.1%)	19 male (42.2%)
Years edentulous	26.0 (20.8) 32, 3 to 45 1 to 58	20.2 (17.7) 14, 5 to 38 1 to 57	23.1 (19.3) 15, 5 to 40 1 to 58
Anxiety (Modified Dental Anxiety Scale)	10.8 (3.8) 11, 8 to 14 5 to 17	11.6 (6.6) 9, 6 to 15 5 to 25	11.2 (5.4) 10, 7 to 14 5 to 25

^a^Values are mean and standard deviation along with median, interquartile range, minimum and maximum for age, number of years edentulous and anxiety

Table 3 presents the clinical and patient rates of oral health quality of life outcomes. No participants refused to take the ‘gummy jelly’ test and we were able to measure the glucose released from all samples. The pattern of results for the ‘gummy jelly’ test did not reflect those of the OHIP-20 or subjective assessment of chewing ability (see Fig. 5.) However, due to differences in batch composition the glucose released at 6 months was not comparable to baseline or 2 month measures.

**Table 3:** Summary statistics for clinical and QoL outcomes

	Mini implants						Conventional implants					
Outcome	Baseline (*n* = 22)	Operation (*n* = 22)	24 h (*n* = 22)	1 week (*n* = 22)	2 months (*n* = 20)	6 months (*n* = 19)	Baseline (*n* = 23)	Operation (*n* = 23)	24 h (*n* = 23)	1 week (*n* = 23)	2 months (*n* = 23)	6 months (*n* = 22)
Glucose Mean (sd) Median (IQR)	145.18 (54.00)				193.95 (86.84)	198.84 (111.76)	185.35 (81.02)				189.21 (87.11)	187.86 (74.21)
	132 (109 to 184)				172 (152 to 204)	170 (99 to 241)	164 (126 to 230)				161 (138 to 208)	180.5 (135 to 246)
Pain^a^ Mean (sd) Median (IQR)		6.5 (5.5)	3.05 (2.6)	16.8 (19.38)				11.58 (14.6)	4.3 (3.2)	38 (32.2)		
		4 (3 to 10)	3 (0 to 5)	6.5 (2 to 27)				5 (3 to 15)	3 (1 to 7)	37 (7 to 69)		
Analgesic consumption		15/22 (68.18%)		8/22 (36.36%)				20/23 (86.96%)		16/23 (69.57%)		
OHIP-20^b^Mean (sd) Median (IQR)	76.5 (20.1)				66.5 (28.3)	40.9 (18.1)	83.3 (23.1)				79.6 (24.9)	56.2 (29.6)
	78.5 (60 to 92)				65 (43 to 80)	36 (25 to 58)	81 (64 to 100)				82 (58 to 105)	43 (29 to 81)
Affects general health^c^	14/22 (63.6%)				5/20 (25%)	5/19 (26.3%)	12/23 (52.2%)				7/23 (30.4%)	4/22 (18.2%)
Implant Failure					1	0					0	0

^a^Pain assessed at the end of surgery (100 mm VAS), 24 h post surgery (0 to 10 VRS) and 1 week (100 mm VAS)

^b^OHIP-20 has 20 items that are rated on six-point Likert type scales (range 1 = never to 6 = always). The total score of the scale ranges from 20 to 120 points, with lower scores indicating better oral health-related quality of life (OHRQoL). Total scores were calculated without item weighting

^c^One of the questions in the satisfaction questionnaire was whether the participants felt that their oral condition impacted on their general health

**Fig. 3:**
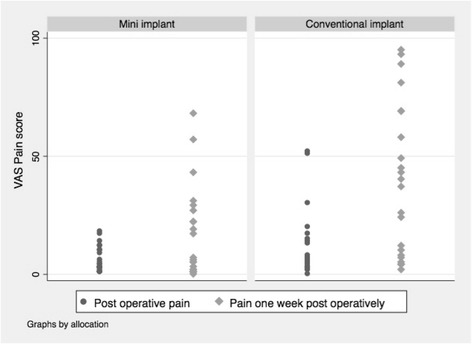
Distributions of responses to pain outcome

Pain VAS scores recorded immediately post-op and after 1 week post-op are presented in Fig. 3. The range of scores was wider in the conventional implant group compared to the mini implant group; this difference in distribution was most notable 1 week post-operatively.

Clinician and patient reported analgesic consumption, at face value, was also higher in the CI group compared to the MI group during the surgical procedure and at 1 week post-surgery.

**Fig. 4:**
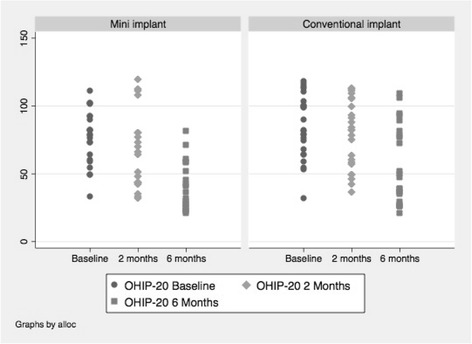
Distributions of responses to OHIP-20 outcome

The results of the OHIP-20 scores are presented in Fig. 4. There was downward trend in OHIP QoL scores in both groups at 6-month follow up compared to baseline suggesting an improvement in oral health related quality of life.

**Fig. 5:**
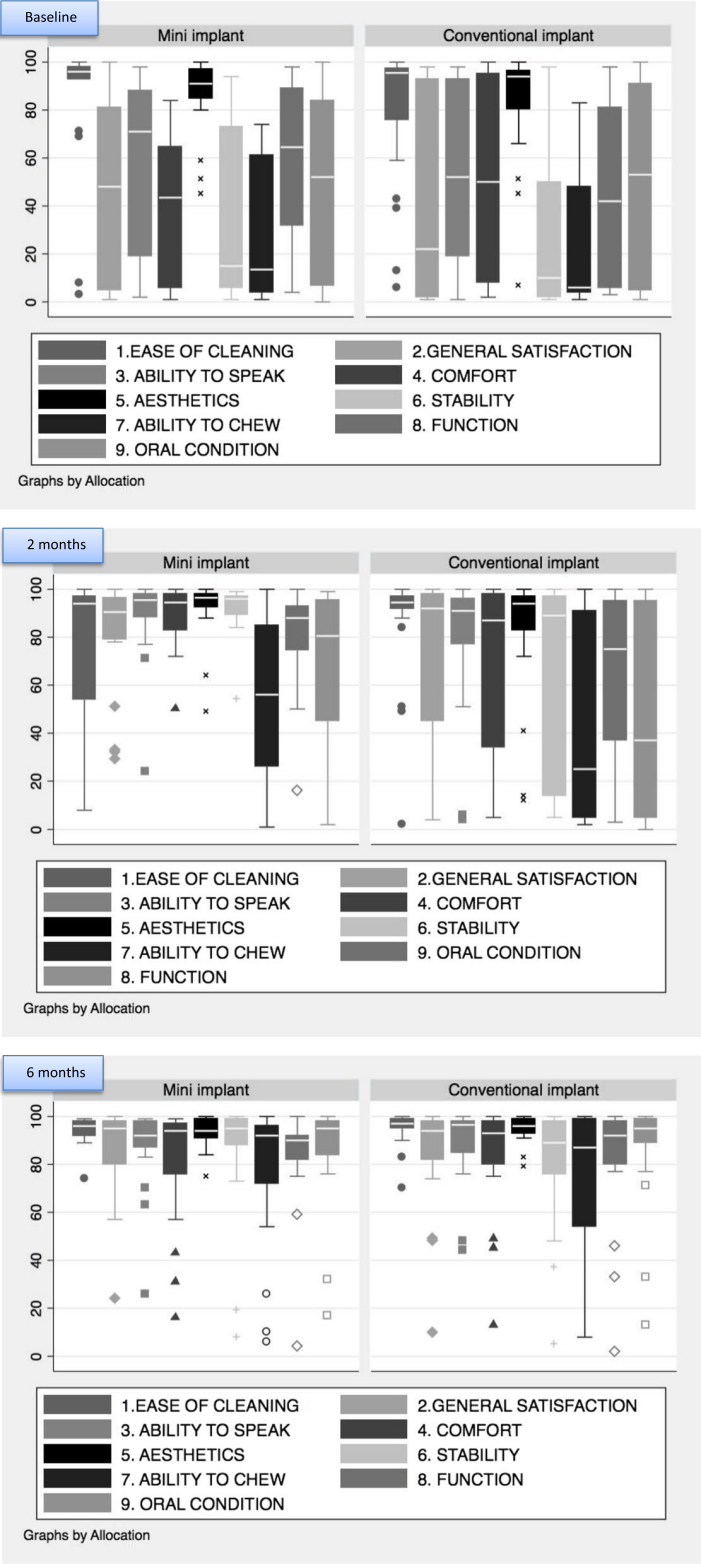
Distributions for each domain of the prosthesis assessment questionnaire (Baseline, 2 months, 6 months)

The denture satisfaction questionnaire records different aspects of patient satisfaction with their prosthesis using a 100 mm VAS. The scales are anchored by the extremes of potential responses (e.g., not at all satisfied to extremely satisfied). Higher values indicate a positive response and improvement. The distributions of responses for each domain of the prosthesis assessment questionnaire are shown in Fig. 5.

There were no adverse events reported in the MI group. CI group had 4 adverse events (8.9%). One was due to pain, and three due to infection. All were treated medically with painkillers and antibiotics as appropriate. One patient died during the follow-up period, from a condition unrelated to treatment. No other Serious Adverse Events were recorded.

There was a total of 74 unscheduled visits in 45 patients (mean 1.7, range 0 to 6): In the MI group there were 23 implant-related visits, due primarily to problems with O-ring attachments, and 18 prosthesis-related visits; in the CI group there were 13 implant-related visits, 17 prosthesis related visits and 3 visits for other reasons.

The costs of the MI and C- groups are presented in Table 4. Mean NHS costs observed over the trial for MIs were lower than that for CIs (column 1 of Table 4, £296 vs. £688). The MI group had lower observed mean staff costs (£37 vs. £88), equipment and consumable costs (£181 vs. £532), and medication costs (£0.23 vs. £4.24). The MI group had higher observed mean unscheduled visits costs (£78 vs. £63) (a detailed analysis of all costs recorded and methods used to generate costs are contained in the Additional file 1).

**Table 4:** Cost information

	NHS cost	Patient cost	NHS and patient cost
Mini implants			
Mean	296.09	193.22	480.60
Sd	126.93	119.91	249.89
Count	22	7	7
Median	237.33	178.58	389.19
IQR	218.19 to 370.16	112.70 to 248.09	330.81 to 635.33
Range	194.41 to 736.72	62.73 to 430.23	277.90 to 984.82
Conventional implants			
Mean	688.30	155.57	813.99
Sd	124.17	70.68	93.62
Count	23	8	8
Median	655.31	157.89	823.17
IQR	628.13 to 687.45	106.73 to 198.26	750.10 to 860.51
Range	585.75 to 1189.69	54.07 to 264.72	676.53 to 967.84
Overall			
Mean	496.56	173.14	658.41
Sd	233.90	95.07	246.54
Count	45	15	15
Median	607.63	170.26	696.81
IQR	239.68 to 656.03	112.70 to 222.36	389.19 to 847.45
Range	194.41 to 1189.69	54.07 to 430.23	277.90 to 984.82

There was a higher mean patient cost observed for the MI group (£193 vs. £156) (column 2 of Table 4). Missing data was evident in most patient costs; most patients reported the same costs at each visit and were perplexed that the same questions were asked at each visit. The compounding nature of missing data (since there are three instances of follow ups and numerous unscheduled visits) resulted in a final complete cost sample of 15 patients. Of these, the mean observed costs of MI were lower than that for CI (£481 vs. £814).

Table 5 presents the results of EQ-5D and SF-6D questionnaires at baseline and at 2 and 6 months assessment. A higher score represents higher health-related QoL (HRQoL). Data was complete in the sense that no missing data was apparent aside from those patients who did not complete the trial. At face value mean baseline EQ-5D and SF-6D scores were lower for the CI group compared to the MI group. Mean EQ-5D and SF-6D scores for both groups showed little variation at 6 months follow-up compared to baseline. Comparisons of changes in EQ-5D and SF-6D scores from baseline to 6 months assessment for those completing the trial suggest reductions in EQ-5D score for both implant groups. For SF-6D scores there appears to be higher mean SF-6D scores at 6 months compared to baseline for the CI group and a reduction in mean scores for the MI group.

**Table 5:** Health-Related Quality of Life HRQoL measures

	Mini implants				Conventional implants			
Outcome	Baseline (*n* = 22)	2 months (*n* = 20)	6 months (*n* = 19)	Baseline (complete cases, *n* = 19)	Baseline (*n* = 23)	2 months (*n* = 23)	6 months (*n* = 22)	Baseline (complete cases, *n* = 22)
EQ-5D Mean (sd) Median (IQR)	0.7224 (0.3276)	0.7289 (0.3598)	0.7127 (0.2968)	0.7146 (0.3524)	0.6401 (0.3639)	0.5840 (0.4489)	0.6241 (0.3715)	0.6458 (0.3715)
	0.7960 (0.7250 to 1.0000)	0.8050 (0.6380 to 1.000)	0.7270 (0.6200 to 1.0000)	0.7960 (0.7250 to 1.0000)	0.6560 (0.5160 to 1.0000)	0.7960 (0.0880 to 1.0000)	0.8220 (0.1890 to 1.0000)	0.6905 (0.5160 to 1.0000)
SF-6D Mean (sd) Median (IQR)	0.7153 (0.2060)	0.6844 (0.2465)	0.6943 (0.2404)	0.7220 (0.2149)	0.6714 (0.2158)	0.6720 (0.2130)	0.7263 (0.2117)	0.6767 (0.2193)
	0.7670 (0.6150 to 0.9220)	0.7580 (0.4055 to 0.8715)	0.8000 (0.4070 to 0.8800)	0.8000 (0.6150 to 0.9220)	0.7190 (0.4530 to 0.8630)	0.6900 (0.4670 to 0.8630)	0.8000 (0.4670 to 0.9220)	0.7645 (0.4530 to 0.8630)

Values are mean, standard deviation, count, median, interquartile range

## Discussion

### Overview of the findings

This paper reports the findings of a National Institute for Health Research (NIHR)-funded pilot trial, the main purpose of which was to inform the design and conduct of future trials. Conventional implants have a long history and their clinical outcomes have been well documented [46–50]. Over the last 30 years, successive Adult Dental Health Surveys [51] have shown that the number of edentulous patients has fallen dramatically. The inability to retain a mandibular denture is debilitating, with dental need falling across the whole population a case could be made in the UK for NHS resources to be redirected to address the significant needs of this patient group. Mini implants could offer the NHS an effective and affordable means of providing care for this group of patients as a first line course of treatment. But to make a convincing case a high-quality, large, multi-centre trial with adequate follow up is required.

The primary aim of this study was to assess the feasibility of a conducting a large surgical randomised controlled trial as proposed in a population of patients who can benefit from an implant retained lower denture. The identified key areas of uncertainty to be explored in this trial focused upon the processes of recruitment and retention of participants, choice of clinical, QoL and cost outcome measures and their method of capture.

Our concerns over the willingness of this cohort to be randomized to different surgical options proved to be unfounded. We reached our required sample size within the pre-specified recruitment period, and very few eligible patients declined to take part in the trial because of a strong patient preference or unwillingness to be randomised. These results suggest that recruitment of participants through a major, secondary referral centre is feasible. Moreover, attrition over the 6 months duration was minimal (43 out of 46 patients (93.5%) completed the trial) with only four adverse events reported, all minor in nature and related to the surgery. However, follow up in this pilot study was limited to 6-months; in trials with a predominantly elderly sample some attrition from mortality unrelated to the intervention can be expected.

Trial participants were accepting of the test of masticatory function (‘gummy jelly’ test [52]) as evidenced by the number of samples provided, but the lack of consistency in measurement over time suggests that this measure is underdeveloped. Further development and evidence of repeatability and reliability is needed before the test could be used with confidence in a clinical trial.

All participants provided responses to pain outcome at all time-points. The data suggests we could expect to find a large difference in short-term post-operative pain scores between the groups in a definitive trial. Descriptively, the pain scores of participants in the CI group were substantially higher than participants in the MI group at 24 h and 7 days post-surgery. It would seem that 7 days post-surgery would be the optimal time for measurement and little is to be gained from measuring at both 24 h and 7 days.

Our Public Patient Involvement (PPI) group strongly recommended QoL as a primary outcome measure for any future trials in this area. Adherence to the NICE guidance for health technology appraisal requires health effects to be expressed in Quality Adjusted Life Years [43]. A key finding from our research is that the components of the generic EQ-5D and SF-6D HRQoL measures seem to show poor responsiveness to oral health-related changes in quality of life and many participants queried the relevance of the questions in these measures to an oral health intervention. A full trial may wish to utilise only one of the HRQoL measures, our data suggests SF-6D may provide some variability in the different implant groups but the EQ-5D is the preferred measure of HRQoL to aid in consistency when comparing the health effects across appraisals and so should be the measure of choice to use in future trials [43].

Data indicated that the OHIP-20 measure [3, 53, 54] and the individual items of the Assessment of Prosthesis scale [7] were sensitive to changes before and after placement of implants. The Assessment of Prosthesis scale performed as expected, with improvements between pre and post implant placement in both groups, but no apparent distinction between the two groups regarding ease of general satisfaction, cleaning, aesthetic and oral conditions. Satisfaction level was high in both groups after fitting the implants. Ability to speak, the level of comfort, stability of the dentures, perception of the chewing ability and function showed similar improvement in both groups pre and post implant placement. From a trials design perspective the lack of a single summary statistic to summarise each individual’s response means that this instrument in its entirety cannot be used to calculate a trial sample size but the individual items of this measure particularly, subjective assessment of chewing ability, can provide valuable information as a secondary outcome measure in the absence of well-validated objective measures.

Responses to the OHIP questionnaire illustrated the expected improvement in oral health-related QoL and the pattern of responses in each group was very similar. This could indicate either that the effects of the interventions are indeed very similar, or that the instrument is insufficiently sensitive to detect any differences between the groups. Our view is that survival should be the primary outcome measure of a future multi-centre trial. This is the primary outcome measure of the majority of implants trials reported in Cochrane systematic reviews [42, 55–59] and will be the key determinant of clinical and economic outcomes. The literature suggests that mini implants have a lower survival rate than conventional implants [29, 34, 38, 40, 60, 61] but from the data we present seem less expensive to place and also to replace if they fail. Studies [34, 60, 61] also suggest that if an implant is going to fail it is likely to happen in the first 2–3 years, suggesting that follow up period for future trials could be confined to 3 years, which would help contain costs. Although in this pilot trial follow-up was limited to 6 months, implant failure was rare, with only one failure of a single implant in the mini implant group and no failures in the conventional implant group. A subsequent audit of longevity of mini implants placed, involving all cases completed in University Dental Hospital of Manchester Restorative Department over a 92-month period, indicates that most failures occur in the first 2–3 years following placement. A low event rate would mean that a substantial number of participants would be required to achieve adequate power for a Randomised Controlled Trial (RCT) if implant failure is the primary outcome.

The pilot trial provided important information with regard to the health economics of implant-retained dentures. Any difference in costs appears to be driven by the differences in implant (materials) costs and staff costs (reflected in staff time). Large differences in staff costs, equipment and consumable costs were evident between the groups but little difference in medication costs. The number of unscheduled visits amount to 41 for the mini implant group and 33 for the conventional implant group. Our PPI group recommended a shorter, single measure of indirect (patient-borne) costs should be used in a full trial. Unscheduled visit costs to the NHS and medication costs did not substantially differ between groups and neither did patient costs (travel, time off work etc.). This strongly suggests that in future studies measurement of costs should be mainly restricted to direct costs of treatment, whether that is borne by the state, insurance companies or individual patients. One issue that our PPI group raised, which is important for future trials to consider, is the costs of ongoing maintenance of implants in primary care. Patients reported difficulty in finding providers of ongoing care and also the high costs of maintenance both of which need to be measured and factored into health economic analyses of future trials.

### Strengths and limitations

This trial had narrow inclusion criteria; only patients with the most atrophic mandibles were invited to participate (Cawood and Howell Class V or VI [41]). However, in the screening process summarised in the CONSORT flow diagram (Fig. 1.) of the 99 patients who were ineligible for inclusion 48 were excluded because their mandibular ridge was Class IV or better, but these patients still had problems retaining their lower denture. Also there is evidence [62] to demonstrate that placement of implants slows down bone resorption and so reduces the clinical problems faced by surgeons and prosthodontists. These findings strongly suggests that many more patients could benefit from implants than patients with Class V or VI ridges and points to the need for widening inclusion criteria in future large, multi-centre pragmatic trials. Broadening inclusion criteria exclusion criteria could result in a higher recruitment rate and increase applicability of findings.

Other than implant failure and chewing ability, we limited our measurements to patient reported outcomes. The rationale behind this choice was that we wanted to ensure that in addition to clinically important outcomes, we captured outcomes that were important to patients. There was uncertainty as to what patient reported outcomes would be acceptable and useable with elderly people in a secondary care setting. Conversely, there is a consensus on clinically important outcomes such as failure of the denture superstructure retained by the implants (total replacement or refurbishment needed), quality and quantity of peri-implant bone. Such measurements are routinely taken in secondary care (where the full trial would be undertaken) and so uncertainty surrounding the use of these outcome measures within an RCT is minimal.

Although the standard practice is the placement of four or more mini implants in the interforaminal region [26, 28–30, 32, 36], this study trialled the use of two, which has been shown in clinical case studies and in previous studies [25, 31] to provide equivalent retention to the recommended four. A recent RCT compared 4 mini implants with 2 mini implants with 2 conventional [63]. Further trials are required to assess the costs effectiveness of 2 versus 4 implant retained lower dentures. Although it could be argued that if mini implants fail they can be readily and cheaply replaced, however this assumption needs to be robustly evaluated in future trials.

This was a pilot trial; its purpose was to ascertain whether a full trial would be feasible and justified and to collect information to inform the design and conduct of subsequent trials. Our trial has shown that a full evaluation of ‘which is more cost-effective, mini implants or conventional implants to retain a lower denture?’ using the proposed design and methods we employed is feasible. The data suggest that mini implants are cheaper, have less post-operative pain and less complications than conventional implants whilst producing similar QoL improvements. However, the follow up period was too short to assess implant survival and long term cost effectiveness. The study was also restricted to one site and to have greater external validity, multi-centre trials are required in different populations with different care providers.

## Conclusions

Recruitment and retention of sufficient, eligible patients to a trial within a realistic timescale is possible. There are limitations in using generic and ‘disease-specific’ QoL measures to evaluate effectiveness in this context. Implant survival is important for patients and has a major impact on clinical effectiveness and costs and should therefore be considered as a primary outcome measure. Outcomes should be assessed at 7 days post operatively (pain) and 6 monthly intervals (implant survival) following implant placement.

## Additional file

Additional file 1: CIMIDENT HE Report BMC Oral Health Appendix. Health Economics Analysis. Methods and analysis of costs for the two implant procedures and subsequent comparison. (DOCX 83 kb)

## Abbreviations

CI: Conventional Implants
EQ-5D: Euroqol-5D questionnaire
HRQoL: Health-Related Quality of Life
IQR: Interquartile range
MI: Mini implants
NHS: National Health Service
NICE: National Institute for Health and Care Excellence
NIHR: National Institute for Health Research
OHIP: Oral Health Impact Profile
OHRQoL: Oral health-Related Quality of Life
PPI: Public Patient Involvement
QoL: Quality of Life
RCT: Randomised Controlled Trial
RfPB: Research for Patient Benefit
SD: Standard deviation
SF: Short Form
TTO: Time Trade-Off
VAS: Visual Analogue Scale
VRS: Verbal Rating Scale
WHO: World Health Organisation
