# Supplementary material for: A pilot randomised controlled trial evaluating mini and conventional implant retained dentures on the function and quality of life of patients with an edentulous mandible
Source: BMC Oral Health. 2017 Feb 15;17:53. doi: 10.1186/s12903-017-0333-1 (PMC5310054; doi:10.1186/s12903-017-0333-1)
Supplement: Additional file 1: — CIMIDENT HE Report BMC Oral Health Appendix. Health Economics Analysis. Methods and analysis of costs for the two implant procedures and subsequent comparison. (DOCX 83 kb) [file 12903_2017_333_MOESM1_ESM.docx]

**Health Economics Analysis**

**Methods**

The aim of the feasibility study was to inform the best way to measure costs and outcomes of the two dental implants. The health economics analysis presents the resources recorded and their respective unit costs and compares costed resource use between the two implants. Outcomes included in the health economics piece are those capturing Health-Related Quality of Life (HRQoL). HRQoL was recorded via the SF-12 and EQ-5D in accordance with the National Institute for Health and Care Excellence guidelines for health technology assessment (NICE, 2013) and converted to preference-based measures. The EQ-5D-3L questionnaire responses were converted to EQ-5D index scores scoring (Dolan, 1997), and the responses to the SF-12 were converted to the SF-6D (Brazier and Roberts, 2004). Comparison is made between the EQ-5D and SF-6D preference-based measures recorded in the trial. All analysis was conducted in Stata version 13 (StataCorp, 2014).

**Cost analysis**

We first present costs from the NHS and social care provider perspective, secondary analysis includes costs from the patient perspective this approach approximates a societal perspective. Patient resource use was collected via questionnaires at baseline; one week follow up; two month follow up; and six month follow up. Additional data on resource use was collected via the dental hospital concerning times in treatment and medical equipment utilised. Where possible unit costs were collected using national sources namely: NHS Reference Costs (Department of Health, 2014); Personal Social Services Resource Unit costs (Curtis, 2014); and the British National Formulary (Medicines Complete, 2014). Table A1 provides all unit costs employed in the analysis.

NHS Costs

These costs are directly related to the implant procedure including the operation and any additional healthcare resources related to the procedure. The forms of costs are:

1. Staff costs
2. Equipment and consumable costs
3. Medication costs
4. Adverse events & unscheduled visits
5. Additional healthcare resource use (e.g. hospital and primary care visits)

*Staff costs*

Overheads (management, administration and estates staff, offices, telephones, education, training, supplies and services (clinical and general), and utilities) as well as capital overheads are contained in the unit cost of health and social care (Curtis, 2014). Unit costs also account for sickness absence (are based on working time). For consultants we use the unit cost of a Surgical Consultant (£142 per contract hour, pg.257 of Curtis (2014)):

For Specialist registrars (SpR) we use the unit cost of a Specialist Registrar. We assume the SpR is trained and working a 40 hour week (i.e. is costed at £71 per hour, pg.255 of Curtis (2014)).

For a nurse we use the hourly rate taken from the total NHS cost attributed per annum over the trial. The total NHS cost was £3,217 in year 1 and was for 10% of the nurse’s time. Assuming 52 weeks a year (i.e. that the salary covered holiday and public holiday entitlement), and under a 5 day week at 37.5 hours this covers 3.75 hours multiplied by 52 – 195 hours. The hourly rate for the nurse is thus £3,217/195=£16.50. Note that estate costs are partly incorporated under the consultant and SpR hourly rates.

The cost of staffing is determined by the time taken for a procedure as recorded at the dental hospital. A dental nurse and consultant conducted all procedures and SpR all unscheduled procedures not involving surgery. Time at hospital was not recorded for follow up so no staff costs are applied for follow up. Mini implants procedures had an average lower mean procedure time (13.91 minutes for mini implants and 33.39 minutes for conventional implants) resulting in relatively lower staff costs.

*Equipment and consumables costs*

The consumable unit costs for conventional implants are £515.23 and for a mini implant £159.48 (see Table S1). The unit costs of all additional equipment used at operation (both consumables and reusable equipment) are provided in Table SA1, the unit costs for these consumables and equipment were obtained from the dental hospital.

Table S1 Implant unit costs (consumables)

|  | Mini (3M) | Mini (3M) per unit | Conventional (Astra)* | Conventional (Astra) per unit |
| --- | --- | --- | --- | --- |
| Implants | £68.25 | £68.25 | £224.00 | £224.00 |
| Ball abutment | N/A | £0.00 | £120.50 | £120.50 |
| Ball abutment housing | £25.20 | £25.20 | £44.00 | £44.00 |
| ‘O’ ring (pack of 10)** | £21.00 | £2.10 | N/A | £0.00 |
| Clix insert** | N/A | £0.00 | £19.00 | £19.00 |
| Surgical kit (all autoclavable and reusable) | £46.20 (inc drills) | £46.20/20=£2.31 | £579.50 (exc drills)  Drills:  (£10.50+(£38.50x2))  Ratchet wrench £167.50  Driver handle £41.50  Restorative kit £219  Restorative wrench £251  Ball wrench £148.50 | £579.50+  £87.50+  £167.50+  £41.50+  £219.00+  £251.00+  £148.50  =£1494.50/20  =£74.73 |
| Lab analogue*** | £24.15 | £24.15 | £15.50 | £15.50 |
| Pick up coping (not used) | £37.47 | £37.47 | £17.50 | £17.50 |
| Total |  | £159.48 |  | £515.23 |

*Dental hospital gets 12.5% discount off these prices, not applied to reflect market value

**1 ‘O’ ring/clix needed per implant.

***costs borne by lab

*Additional healthcare resources*

These are not directly accrued in the application of the treatment, but stem from the treatment. These costs cover any adverse events (healthcare use) and medication.

Outpatient oral surgery

No additional hospital or community care related to the procedure were observed in the trial, this implies that no additional healthcare costs outside of the dental hospital were incurred.

Adverse events

There were 4 recorded adverse events all located in the conventional implant arm. 1 due to pain, 3 due to infection. None were coded as serious. 3 were mild in pain, 1 moderate. All 4 had treatment involving mediations. Medications were costed in accordance with Table SA1.

Unscheduled visits

Of the 45 patients 13 had no unscheduled visits. 32 therefore had unscheduled visits. Total volume of unscheduled visits amount to 41 for mini implants and 33 for conventional implants (Table SA2). Unscheduled visits were more prominent in those with mini implants.

The method of costing unscheduled visits was the same as for scheduled visits: staff costs were calculated from data on the time a patient was in treatment (time in building was not recorded so patient time was recorded for treatment time only), and consumables were costed in accordance with Table SA1. Unscheduled visits were coded with respect to the main reason for the visit: due to implant, prosthesis, or other. Mean staff time and total NHS costs (staff costs and consumables) by type of visit are reported in Table SA3.

Medication

Medication was reported and assigned to whether this was associated with: operation pain, adverse event pain, or infection. The list of associated medications are provided in Table SA4. There are multiple instances of the same medication (for different days rather than distinct episodes). The unit costs were obtained via the British National Formulary as at November 2014 (Medicines Complete, 2014). The total number of tablets were derived from the reported dosage and days taken, this was then multiplied by the cost per tablet. Table A5 gives the mean medication costs at operation, for adverse events, and infection. Adverse events and infection medications are exclusively amongst conventional implants.

*Total NHS Cost*

Total NHS costs without and with unscheduled visit costs are provided in Table S2.

Table S2 Total NHS costs

|  | Staff | Equipment and consumables | Medication | NHS cost without unscheduled | Unscheduled visits | NHS with unscheduled |
| --- | --- | --- | --- | --- | --- | --- |
| Mini implants | 36.74  26.60  [13.21,121.52] | 181.15  0.39  [179.70,181.65] | 0.23  0.27  [0,0.89] | 218.13  26.60  [194.41,302.72] | 77.97  124.67  [0.00,536.84] | 296.09  126.93  [194.41,736.72] |
| N (missing) | 22 (0) | 22 (0) | 22 (0) | 22 (0) | 22 (0) | 22 (0) |
| Conventional implants | 88.21  18.49  [55.48,132.08] | 532.41  4.57  [528.31,540.76] | 4.24  12.60  [0,59.61] | 624.86  20.32  [585.75,670.05] | 63.44  121.52  [0.00,579.14] | 688.30  124.17  [585.75,1189.69] |
| N (missing) | 23 (0) | 23 (0) | 23 (0) | 23 (0) | 23 (0) | 23 (0) |

Prices are: mean, standard deviation, and minimum and maximum

Patient Costs

Patient costs include: transport costs (parking, petrol, public fares, and time travelled); time spent in the dental hospital; and accompanying person costs.

*Transport*

There are four records where detailed information is asked with regards to transport and time travelled: Operation; 1 week follow up; 2 month follow up; and 6 month follow up. In the first stage we calculate the cost for parking from patient reported costs (this is zero for disabled badge holders). In the second stage we calculate the public transport reported cost. We assumed this was a one way fare and multiplied by two the cost of transport. This could be an issue if patients travelled peak time and had return fares discounted (free transport). The third stage we calculate the mileage travelled. This is then recoded as zero for public transport and multiplied by the price per litre of unleaded fuel. Price per litre of fuel is obtained from the AA (AA, 2014) and converted to mile by Fuel Calculator (Fuel Economy, 2015). This gives a price per mile of £0.16. It was assumed the reported mileage was for a one way journey. Total mileage was then multiplied by two and the unit cost per mile for fuel. In the fourth stage we calculate the value of time taken travelling. This is multiplied by two (assuming the same time is taken for a round trip). For those working we apply a value of the median hourly earnings of £11.54 (unless reported). For those not working we apply a value of time of £6.04. The non-working value was obtained via the Department for Transport WebTAG Table A1.3.1 (Department of Transport, 2014) whereby the market price for those not working of the value for time for other (as opposed commuting) was used.

*Productivity*

We take the wage lost as reported by the individual, and where annual leave is taken we multiply the average wage of £11.54 by 7.5 hours. There was one patient in the mini implant arm and one patient in the conventional implant arm who report annual leave at operation, the mini implant patient reported a lost wage of £100 at operation (as this patient also reported annual leave – we took the highest loss (wage at £100.00)). At 1 week follow up one patient in the mini implant arm reported annual leave. At 2 month follow up one patient in the mini implant arm reported a lost wage of £120. At 6 month follow up one patient in the mini implant arm reported a lost wage of £80.

In addition to time spent travelling to and from the dental hospital patients spend time at the hospital waiting and during the procedure. Patient times have been recorded by the dental nurse. Total time in building is measured in minutes and valued in accordance to the national median hourly wage (£11.54) for those employed and £6.04 for those not working.

*Family/accompanying persons cost*

There were 31 patients with an accompanied person at operation, 29 at 1 week follow up, 24 at 2 month follow up (and 1 missing), and 22 at 6 month follow up (and 3 missing). The accompanying person was usually the patient’s spouse (61.29%, 55.17%, 58.33%, and 59.09% respectively). Transport costs were reported in the same manner as that for the patient though additional details were asked regarding the time and distance travelled to pick up the patient (if applicable).

For productivity losses we follow the same approach as that for patients: where wages or annual leave are reported we take this value or £11.54*7.5 (whichever is greater). 4 (/31) accompanying people at operation were in employment, 3 (/29) at one week follow up, 4 (/25) at 2 month follow up, and 3 (/22) at 6 month follow up. In the questionnaire where respondents reporting wage losses stated only whether there was a loss in wage, a value was assigned of £11.54*7.5.

*Unscheduled visits*

Each unscheduled visit had a respective Health Economics Questionnaire completed. The total cost of unscheduled visits is presented here as opposed to cost by visit number. The methodology to generate the total patient and accompanying person costs is identical to that of scheduled visits.

*Total patient costs*

To calculate the total reported costs from the Health Economics Questionnaire for transport and productivity we sum all costs, but make the following amendments:

- For parking costs we take the maximum of the patient reported parking cost and the accompanying person parking cost. This assumes that both patient and accompanying person travelled in the same car
- For mileage we take the maximum reported mileage from the patient and accompanying person

For wages and public transport we take the full mentioned cost for both patient and accompanying person. For time taken we take the accompanying person and add this to the patient since both have taken time to travel, we also add to this the time the accompanying person reports as travelling to pick the patient up and assume this is the same on return, where a wage is reported as lost then all time travelling is replaced with zero for that respective person. Time at hospital (recorded by the dental team) is multiplied by two where an accompanying person is in place and no productivity loss from work has been applied.

The components of patient costs are provided in Table S3.

Table S3 Total patient and accompanying person cost breakdown for travel and related productivity (Health Economics Questionnaire)

|  | Parking | Public transport | Mileage | Time  travelled | Wages and annual leave lost | Time spent at operation | Public transport^ | Time travelled^ | Pick up time travelled^ | Wages and annual leave lost^ | Unscheduled visits |
| --- | --- | --- | --- | --- | --- | --- | --- | --- | --- | --- | --- |
| Mini implants | 4.33  5.83  [0,16] | 7.06  24.65  [0,104] | 14.04  13.57  [0,41.60] | 12.49  15.59  [0,56.37] | 17.57  82.41  [0,386.55] | 11.70  5.71  [0,21.95] | 7.06  24.65  [0,104] | 12.49  15.59  [0,56.37] | 1.75  5.40  [0,20.13] | 13.77  48.86  [0,216.38] | 48.40  75.89  [0,307.50] |
| N (missing) | 21 (1) | 18 (4) | 22 (0) | 17 (5) | 22 (0) | 22 (0) | 18 (4) | 17 (5) | 14 (8) | 22 (0) | 22 (0) |
| Conventional implants | 6.75  6.49  [0,16] | 7.50  33.54  [0,150] | 14.65  21.98  [0,102.72] | 23.46  22.56  [0,61.41] | 3.76  18.05  [0,86.55] | 16.51  6.48  [0,28.19] | 7.50  33.54  [0,150] | 23.46  22.56  [0,61.41] | 3.59  5.76  [0,16.11] | 3.76  18.05  [0,86.55] | 26.45  31.15  [0,113.14] |
| N (missing) | 22 (1) | 20 (3) | 22 (1) | 18 (5) | 23 (0) | 23 (0) | 20 (3) | 18 (5) | 16 (7) | 23 (0) | 23 (0) |

Prices are: mean, standard deviation, and minimum and maximum
^accompanying person costs
Note: an individual can have multiple unscheduled visits, hence the high unscheduled maximum cost

NHS and patient cost

Total NHS, patient, and NHS and patient costs are provided in Table S4.

Table S4 Total NHS & patient costs

|  | NHS with unscheduled | Patient | NHS and patient |
| --- | --- | --- | --- |
| Mini implants | 296.09  126.93  [194.41,736.72] | 193.22  119.91  [62.73,430.23] | 480.60  249.89  [277.90,984.82] |
| N (missing) | 22 (0) | 7 (15) | 7 (15) |
| Conventional implants | 688.30  124.17  [585.75,1189.69] | 155.57  70.68  [54.07,264.72] | 813.99  93.62  [676.53,967.84] |
| N (missing) | 23 (0) | 8 (15) | 8 (15) |

Prices are: mean, standard deviation, and minimum and maximum

Cost analysis summary

*NHS cost:*

A difference in the cost of mini and conventional implants were observed over the trial (column 1 of Table S4). This comprises of differences in staff costs, equipment and consumable costs, and differences in medication and unscheduled visits (Table S2). All patients had complete costing data for the NHS costs analysis.

*Patient cost:*

Differences in patient costs were observed (column 2 of Table S4) and this looks to be the product of small differences in patient costs for the variety of patient costs reported, though a large difference was observed for time at the dental hospital (Table S3). Missing data was evident in most patient costs, the compounding nature of missing data (since there are three instances of follow ups and numerous unscheduled visits) resulted in a final complete cost sample of 15 patients.

*NHS and patient cost:*

A difference in the total (NHS and patient) costs of mini and conventional implants was observed over the trial for the 15 patients with complete cost data.

*Missing patient costs supplementary analysis:*

Using only the first reported responses to the patient questionnaire and applying these to subsequent visits would result in a sample of 33 patients with ‘complete’ data in the NHS and patient perspective analysis. The mean NHS and patient costs are provided in Table S5. A difference in total (NHS and patient) costs is found and similar patient costs. Consideration is needed into the validity and value of the patient questionnaire at subsequent visits for any future trial.

Table S5 Comparison of costs

|  | NHS | Patient | NHS and patient | Patient (forward filled) | NHS and patient (imputed) |
| --- | --- | --- | --- | --- | --- |
| Mini implants | 296.09  126.93  [194.41,736.72] | 193.22  119.91  [62.73,430.23] | 480.60  249.89  [277.90,984.82] | 225.63  149.35  [54.74,611.72] | 525.45  210.89  [305.54,1098.36] |
| N (missing) | 22 (0) | 7 (15) | 7 (15) | 17 (5) | 17 (5) |
| Conventional implants | 688.30  124.17  [585.75,1189.69] | 155.57  70.68  [54.07,264.72] | 813.99  93.62  [676.53,967.84] | 217.24  119.02  [71.78,456.96] | 915.06  194.15  [682.12,1439.93] |
| N (missing) | 23 (0) | 8 (15) | 8 (15) | 16 (7) | 16 (7) |

Prices are: mean, standard deviation, and minimum and maximum

**Health Outcomes**

The health economics piece will assess the frequency and changes in EQ-5D and SF-12.

***EQ-5D***

The time trade-off (TTO) measurement for weighting the EQ-5D-3L was taken as this is the recommended utility weight advocated by NICE (NICE, 2013). The TTO weights are obtained from Dolan (1997). Baseline, 2 month, and 6 month EQ-5D scores are provided in Table S6.

The mini implant group have an average HRQoL greater than those patients receiving conventional implants, at baseline. On average, the mini implant group appear to have a slight improvement in HRQoL at 2 month follow up, whilst the conventional implant group have a slight reduction. Between 2 and 6 month follow up the mini implant group have a slight reduction in HRQoL while the conventional implant group have a slight improvement.

Table S6 EQ-5D summary statistics

| EQ-5D | Baseline | 2 months | 6 months | Complete data |
| --- | --- | --- | --- | --- |
| Mini implant  Mean  Sd  Count  Median  Iqr  Range | 0.7224  0.3276  22  0.7960  0.7250 to 1.0000  -0.0740 to 1.0000 | 0.7289  0.3598  20  0.8050  0.6380 to 1.0000  -0.3190 to 1.0000 | 0.7127  0.2968  19  0.7270  0.6200 to 1.0000  -0.0740 to 1.0000 | 2 months:  2 missing  6 months:  3 missing |
| Conventional implant  Mean  Sd  Count  Median  Iqr  Range | 0.6401  0.3639  23  0.6560  0.5160 to 1.0000  -0.1810 to 1.0000 | 0.5840  0.4489  23  0.7960  0.0880 to 1.0000  -0.3190 to 1.0000 | 0.6241  0.4356  22  0.8220  0.1890 to 1.0000  -0.1810 to 1.0000 | 2 months:  0 missing  6 months:  1 missing |
| Overall  Mean  Sd  Count  Median  Iqr  Range | 0.6804  0.3452  45  0.7270  0.5160 to 1.0000  -0.1810 to 1.0000 | 0.6514  0.4117  43  0.7960  0.5160 to 1.0000  -0.3190 to 1.0000 | 0.6652  0.3759  41  0.7270  0.5870 to 1.0000  -0.1810 to 1.0000 | 2 months:  2 missing  6 months:  4 missing |

***SF-12***

The SF-12 is mapped onto the SF-6D (since the SF-6D has HRQoL weights). Weights are obtained from Brazier and Roberts (2004). Baseline, 2 month, and 6 month SF-6D scores are provided in Table 7. The mini implant group have an average HRQoL greater than the conventional implant group at baseline. On average, the conventional implant group appear to have a slight improvement in HRQoL at 2 month follow up, whilst the mini implant group have a slight reduction.

Table S7 SF-6D summary statistics

| SF-6D | Baseline | 2 months | 6 months | Complete data |
| --- | --- | --- | --- | --- |
| Mini implant  Mean  Sd  Count  Median  Iqr  Range | 0.7153  0.2060  22  0.7670  0.6150 to 0.9220  0.3130 to 0.9230 | 0.6844  0.2465  20  0.7580  0.4055 to 0.8715  0.2860 to 1.0000 | 0.6943  0.2404  19  0.8000  0.4070 to 0.8800  0.3130 to 1.0000 | 2 months:  2 missing  6 months:  3 missing |
| Conventional implant  Mean  Sd  Count  Median  Iqr  Range | 0.6714  0.2158  23  0.7190  0.4530 to 0.8630  0.3410 to 0.9220 | 0.6720  0.2130  23  0.6900  0.4670 to 0.8630  0.3410 to 1.0000 | 0.7263  0.2117  22  0.8000  0.4670 to 0.9220  0.4070 to 1.0000 | 2 months:  0 missing  6 months:  1 missing |
| Overall  Mean  Sd  Count  Median  Iqr  Range | 0.6929  0.2098  45  0.7370  0.4880 to 0.8630  0.3130 to 0.9230 | 0.6778  0.2265  43  0.7580  0.4260 to 0.8630  0.2860 to 1.0000 | 0.7114  0.2232  41  0.8000  0.4670 to 0.8800  0.3130 to 1.0000 | 2 months:  2 missing  6 months:  4 missing |

**Conclusion**

The feasibility study informs several important issues with regards to data recording for a full trial. With regards NHS resource use, a difference in cost was found. The difference appears to be driven by the differences in implant costs and time taken (reflected in staffing costs since the same staff members provided both mini and conventional implants). Unscheduled visit costs to the NHS and medication costs did not significantly differ in the study. Data recording was complete in the sense that no missing data affected the sample. However, time at follow up was not recorded.

Patient and accompanying persons costs suffered from poor data recording. This was the result of missing sections of the self reported questionnaire rather than complete null recording. ‘As last time’ was one comment mentioned. Patients reported confusion at being asked this at every visit and this may have manifested in a lack of complete data at follow up(s).

The effects of both implants on HRQoL appear to be small regardless of HRQoL measure. Both HRQoL measures appear to be similar at baseline, though differences in the direction of changes in HRQoL were observed. These results suggest either instrument may be utilised at full trial but either may be unlikely to identify a difference in HRQoL. A full trial may reveal significant change due to sample size, or reinforce the implicit conclusion that the decision of cost-effectiveness between the two implants is one amounting to a cost-minimisation approach.

**Supplementary Appendix Tables**

Table SA 1 Resource use unit costs

| Resource | Source | Unit cost |
| --- | --- | --- |
| Staff costs (All consultations and operative sessions) |  |  |
| Consultant | Curtis (2014) | £142 per contract hour pg.257 |
| SpR | Curtis (2014) | £71 per hour pg.255 |
| Nurse | Trial cost for NHS nurse | £16.50 |
| Time in procedure |  | Timings for cost of staff |
| Equipment and consumables costs | CMFT Management costs for instruments, equipment, and sterilisation  (Actual costs paid): |  |
| Implants |  | A single cost for the different implants (Table 1):  Mini implant: £159.48  Conventional implant: £515.23 |
| LA cartridges | 18.95+vat for 50:  18.95+vat=22.74  /50=0.45 | £0.45 |
| Needle | 20.39 for 100:  20.39/100=0.20 | £0.20 |
| Tissue punch | 24.34 for 20:  24.34/20=1.02 | £1.02 |
| Scalpel blade | 33.51 for 100:  33.51/100=0.34 | £0.34 |
| Straight handpiece | 202.49 for 500 use:  202.49/500=0.40+0.46 (sterilisation)=0.88 | £0.86 |
| Straight stones | 9.55 for 100 use:  9.55/100=0.10+0.44 (sterilisation)=0.54 | £0.54 |
| Implant handpiece | 463.69 for 500 use:  463.69/500=0.93+1.35 (sterilisation)=2.28 | £2.28 |
| Suture | 46.92 for 12:  46.92/12=3.91 | £3.91 |
| Gauze | 71.86 for 200:  71.86/200=0.36 | £0.36 |
| Suction tubing | 15.97 for 20:  15.97/20=0.80 | £0.80 |
| Suction tip | 24.90 for 100:  24.90/100=0.25 | £0.25 |
| MPT | 2.99+0.68 (sterilisation)=3.67 | £3.67 |
| Small syringe | 8.95 for 100:  8.95/100=0.09 | £0.09 |
| Light covers | 138.83 for 50:  138.83/50=2.76*2 per patient=5.55 | £5.55 |
| Blue sterile sheets | 87.83 for 40:  87.83/40=2.20 | £2.20 |
| Non-identified prior |  |  |
| Branemark retractor | 51.26+vat:  61.51 for 500 use:  61.51/500=0.12+0.46 (sterilisation)=0.58 | £0.58 |
| Minesota retractor | 17.24+vat:  20.69 for 500 use:  20.69/500=0.04+0.46 (sterilisation)=0.50 | £0.50 |
| Spencer wells | 4.65 for 500 use:  4.65/500=0.01+0.46 (sterilisation)=0.47 | £0.47 |
| Scissors | 23.90 for 500 use:  23.90/500=0.05+0.46 (sterilisation)=0.51 | £0.51 |
| Pros 2 kit | 462.00 for 500 use:  462.00/500=0.92+0.68 (sterilisation)=1.60 | £1.60 |
| Bite paste | 39.06 for 2:  39.06/2=19.53 for 50ml, 10ml used:  19.53/5=3.91 | £3.91 |
| Dappens pot | 2.09 for 50:  2.09/50=0.04 | £0.04 |
| Brush and pressure paste | 44.30 for 200:  44.30/200=0.22 per 15:  0.22/15=0.01 | £0.01 |
| S1 kit | 246.28 for 500 use:  246.28/500=0.49+1.35 (sterilisation)=1.84 | £1.84 |
| S2 kit | 214.97 for 500 use:  214.97/500=0.43+1.35 (sterilisation)=1.78 | £1.78 |
| Spatula | 3.03 for 500 use:  3.03/500=0.01+0.46 (sterilisation)=0.47 | £0.47 |
| Tray handle | 3.80 for 10:  3.80/10=0.38 for 500 use:  0.38/500=0.00+0.44 (sterilisation)=0.44 | £0.44 |
| Edentulous tray | 4.87 for 25:  4.87/25=0.19 | £0.19 |
| DPT | 48.00 | £48.00 |
| Piezo surgical kit | 3750.00 for 500 use:  3750/500=7.5+1.35 (sterilisation)=8.85 | £8.85 |
| Special perio kit | 147.98 for 500 use:  147.98/500=0.30+1.35 (sterilisation)=1.65 | £1.65 |
| Micro surgical kit | 1292.00 for 500 use:  1292/500=2.58+1.35 (sterilisation)=3.93 | £3.93 |
| Sterile water | 1.85 | £1.85 |
| Articulating paper | 8.23 per 200:  8.23/200=0.04 | £0.04 |
| Identified at unscheduled visits: |  |  |
| Amoxicillin | British National Formulary  Nov 2014:  Pg.106  Amoxicillin 500mg capsules, 15, price £1.20 | £1.20 |
| Impregum | 136.23 for 2:  136.23/2=68.12/5 (1/5 tube used)=13.62 | £13.62 |
| Ball abutments | 120.50 | £120.50 |
| Pick up copings | 37.47 (mini); 17.50 (conventional) | £37.47 (mini); £17.50 (conventional) |
| Amox and metro | British National Formulary  Nov 2014:  Amoxicillin:  Pg.106  Amoxicillin 500mg capsules, 15, price £1.20  Metronidazole:  Pg.146  Metronodazole 200mg tablets, 21, price £9.17  =1.20+9.17=10.37 | £10.37 |
| O rings | 2.10 | £2.10 |
| Mini implant analogues and housing | Lab analogue and ball abutment housing: 24.15+25.20=49.35 | £49.35 |
| Yellow clips exchanged for red | 19.00*2=38.00 | £38.00 |
| Tokuso | 102.40 for 80g, 10g used:  102.40/8=12.80 | £12.80 |
| Healing plate made in lab | 29.50 | £29.50 |
| Coe soft | 54.59 for 80g, 10g used:  54.59/8=6.82 | £6.82 |
| Unscheduled visits (A&E, GP, GDP, dental hospital visits) |  |  |
| A&E attendance | Department of Health (2014) | £124 |
| GP attendance | Curtis (2014) | £46.00 |
| GDP attendance | Curtis (2014) | £65 |
| Hospital visits | Department of Health (2014) | Unscheduled first visit: £168.03  Unscheduled subsequent visits: £109.91 |
| Dental hospital unscheduled visit |  | Costed staff and consumables as for scheduled visits |
| Medication |  |  |
| Painkillers | British National Formulary (2014) | See Table SA4 |
| Patient expenditure |  |  |
| Travel costs | Patient questionnaire | Self reported |
| Earnings lost (patient) | Patient questionnaire  Median hourly earnings:  ONS (2014) | National median hourly wage (£11.54) unless specified and greater than £11.54*7.5 hours |
| Earnings lost (relatives) | Patient questionnaire  Median hourly earnings:  ONS (2014) | National median hourly wage (£11.54) unless specified and greater than £11.54*7.5 hours |
| Total time in building |  | Cost for patient (time valued as above) |

* CMFT Management costs for instruments, equipment, and sterilisation (actual costs paid)
sterilisation costs are for either i) a small pack (£0.44); ii) a double pack (£0.46); iii) a paper bag pack (£0.68); or iv) a wrap (£1.35)

Table SA 2 Unscheduled visits by implant type

| Unscheduled visits | Mini implant | Conventional implant | Total |
| --- | --- | --- | --- |
| 0 | 7 | 8 | 13 |
| 1 | 4 | 10 | 14 |
| 2 | 2 | 2 | 4 |
| 3 | 6 | 2 | 8 |
| 4 | 1 | 2 | 3 |
| 5 | 1 | 1 | 2 |
| 6 | 1 | 0 | 1 |
| Total | 22 | 24 | 45 |

Table SA 3 Unscheduled visit time and NHS cost

|  | Staff time (mins and sample) |  |  | Total NHS cost (£ and sample) |  |  |
| --- | --- | --- | --- | --- | --- | --- |
| Visit type | Mini implant | Conventional implant | Total | Mini implant | Conventional implant | Total |
| Implant (conventional) |  | 46.93  13 | 46.93  13 |  | 73.99  13 | 73.99  13 |
| Implant  (mini) | 36.89  23 |  | 36.89  23 | 53.57  23 |  | 53.57  23 |
| Other |  | 35.97  3 | 35.97  3 |  | 42.44  3 | 42.44  3 |
| Prosthesis | 18.39  18 | 20.76  17 | 19.54  35 | 26.84  18 | 24.76  17 | 25.83  35 |
| Total | 28.77  41 | 20.76  33 | 30.41  74 | 41.83  41 | 45.76  33 | 43.59  74 |

Table SA 4 Medication unit (pack) cost

| Drug | Unit cost | Cost per tablet |
| --- | --- | --- |
| Operation |  |  |
| Paracetamol | British National Formulary  Nov 2014:  Pg.154  Paracetamol 500mg tablets, 32, price £1.02 | £0.03 |
| Ibuprofen | British National Formulary  Nov 2014:  Pg.137  Ibuprofen 400mg tablets, 24, price £1.07 | £0.04 |
| Co-codamol | British National Formulary  Nov 2014:  Pg.118  Co-codamol 30mg/500mg tablets, 30, price £1.51 | £0.05 |
| Tramadol | British National Formulary  Nov 2014:  Pg.169  Tramadol 50mg capsules, 30, price £1.24 | £0.04 |
| Codeine phosphate | British National Formulary  Nov 2014:  Pg.119  Codeine 30mg tablets, 28, price £1.64 | £0.06 |
| Adverse event |  |  |
| Paracetamol | As above | £0.03 |
| Co-codamol | As above | £0.05 |
| Amoxicillin | British National Formulary  Nov 2014:  Pg.106  Amoxicillin 500mg capsules, 15, price £1.20 | £0.08 |
| Infection |  |  |
| Metronidazole | British National Formulary  Nov 2014:  Pg.146  Metronodazole 200mg tablets, 21, price £9.17 | £0.44 |
| Amoxicillin | As above | £0.08 |

Table SA 5 Medication cost

|  | Operation | Adverse event | Infection | Total |
| --- | --- | --- | --- | --- |
| Mini implants | 0.23  0.27  [0,0.89] | 0.00  0.00  [0,0] | 0.00  0.00  [0,0] | 0.23  0.27  [0,0.89] |
| N (missing) | 22 (0) | 22 (0) | 22 (0) | 22 (0) |
| Conventional implants | 0.90  0.82  [0,2.49] | 0.78  3.23  [0,15.36] | 2.56  12.29  [0,58.92] | 4.24  12.60  [0,59.61] |
| N (missing) | 23 (0) | 23 (0) | 23 (0) | 23 (0) |

Prices are: mean, standard deviation, and minimum and maximum

**References**

AA. Fuel Price Report. November 2014. <http://www.theaa.com/resources/Documents/pdf/motoring-advice/fuel-reports/november2014.pdf> [Accessed 3rd December 2015]

Brazier, J.E. and Roberts, J. The estimation of a preference-based measure of health from the SF-12. *Medical Care* 2004; 42(9):851-859

Curtis, L. *Unit Costs of Health & Social Care 2014.* PSSRU 2014

Department for Transport. *TAG UNIT A1.3: User and Provider Impacts*. 2014. <https://www.gov.uk/government/uploads/system/uploads/attachment_data/file/427089/TAG_Unit_A1.3_-_User_and_provider_impacts_November2014.pdf> [Accessed 3rd December 2015]

Department of Health*. Reference costs 2013-14*. <https://www.gov.uk/government/uploads/system/uploads/attachment_data/file/380322/01_Final_2013-14_Reference_Costs_publication_v2.pdf> [Accessed 3rd December 2015]

Dolan, P. Modeling valuations for EuroQol health states. *Medical Care* 1997; 35(11):1095-1108

Fuel Economy. Fuel Calculator. Available from: <http://www.fuel-economy.co.uk/calc.html> [Accessed 3rd December 2105]

Medicines Complete. *British National Formulary*. 2014. Available from: <https://www.medicinescomplete.com/about/publications.htm>. <http://www.nhsbsa.nhs.uk/PrescriptionServices/Documents/PPD%20Drug%20Tariff/November_2014.pdf>

[Accessed 3^rd^ December 2015]

National Institute for Health and Care Excellence. *Guide to Methods of Technology Appraisals*. NICE; 2013.

Office for National Statistics. *Annual Survey of Hours and Earnings, 2014 Provisional Results*. Table 1.6a: Hourly pay – Excluding overtime (£) – For all emoployee jobs: United Kingdom, 2014. 2014 <http://www.ons.gov.uk/ons/publications/re-reference-tables.html?edition=tcm%3A77-337425> [Accessed 3^rd^ December, 2015]

StataCorp. *Stata Statistical Software: Release 13*. 2013.College Station, TX: StataCorp LP.
